# Computational Methods for the Identification of Molecular Targets of Toxic Food Additives. Butylated Hydroxytoluene as a Case Study

**DOI:** 10.3390/molecules25092229

**Published:** 2020-05-09

**Authors:** Valentina Tortosa, Valentina Pietropaolo, Valentina Brandi, Gabriele Macari, Andrea Pasquadibisceglie, Fabio Polticelli

**Affiliations:** 1 Department of Sciences, Roma Tre University, 00146 Rome, Italy; valentina.tortosa@uniroma3.it (V.T.); valentina.pietropaolo@gmail.com (V.P.); valentina.brandi@uniroma3.it (V.B.); gabriele.macari@uniroma3.it (G.M.); andrea.pasquadibisceglie@uniroma3.it (A.P.); 2 National Institute of Nuclear Physics, Roma Tre University, 00146 Rome, Italy

**Keywords:** reverse screening, molecular docking, butylated hydroxytoluene, food additives, synthetic phenolic antioxidants, side effects

## Abstract

Butylated hydroxytoluene (BHT) is one of the most commonly used synthetic antioxidants in food, cosmetic, pharmaceutical and petrochemical products. BHT is considered safe for human health; however, its widespread use together with the potential toxicological effects have increased consumers concern about the use of this synthetic food additive. In addition, the estimated daily intake of BHT has been demonstrated to exceed the recommended acceptable threshold. In the present work, using BHT as a case study, the usefulness of computational techniques, such as reverse screening and molecular docking, in identifying protein–ligand interactions of food additives at the bases of their toxicological effects has been probed. The computational methods here employed have been useful for the identification of several potential unknown targets of BHT, suggesting a possible explanation for its toxic effects. In silico analyses can be employed to identify new macromolecular targets of synthetic food additives and to explore their functional mechanisms or side effects. Noteworthy, this could be important for the cases in which there is an evident lack of experimental studies, as is the case for BHT.

## 1. Introduction

Food additives are natural or synthetic substances which are added to food to maintain or improve its quality, safety, freshness, taste, texture or appearance [1]. European Union (EU) laws establish common rules and procedures for the use of food additives [2]. Approved food additives are divided into 26 functional categories [3] and antioxidant agents represent one of the major classes [1]. Antioxidants are defined as: “substances which prolong the shelf-life of foods by protecting them against deterioration caused by oxidation, such as fat rancidity and colour changes” [3]. They can be classified into two groups according to their antioxidant mechanism of action: primary antioxidants are those that directly react with free radicals breaking the chain reaction of oxidation through the donation of hydrogen and the generation of more stable radicals; secondary antioxidants work indirectly through different mechanisms, such as chelation of transition metal ions, oxygen scavenging, regeneration of primary antioxidants, absorption of UV radiation and de-activation of reactive species [4]. Synthetic phenolic antioxidants (SPAs), like butylated hydroxyanisole (BHA; E320) and butylated hydroxytoluene (BHT; E321), are some of the commonly used food additives. These compounds or their combinations are generally recognized as safe by the U.S. Food and Drug Administration [5]. However, their use is not devoid of side effects to human health. Diverse adverse effects have been reported for BHA such as DNA repair failure, genotoxicity, oxidative stress, carcinogenicity, reproductive toxicities, and endocrine disrupting effects [6,7,8]. Although BHT toxicity is still controversial, side effects have been shown, including carcinogenicity [9,10]. The current legislation takes these risks into account indicating a maximum concentration threshold. However, there is a potential cumulative effect due to the presence of the same additive or different ones in more than one product. In this regard, for example, BHT is present in a variety of consumer products including rubber, foodstuff, cosmetics, plastics, mineral oil and fuel additives, pharmaceuticals, animal food and printing inks [8,9]. Indeed, BHT can be detected in seas, rivers, lakes, sediments and organisms [10,11,12]. To date, only three studies have reported the occurrence of SPAs in human samples [8,13]; thus, very little is known about their true dietary intake. The lack of information about human exposure to these antioxidant agents together with the available toxicology studies raise concerns on their biosafety.

In the last years, in silico analyses have been widely applied in the prediction of protein targets of bioactive small molecules to explain the molecular mechanisms of their action in different diseases [14,15]. Reverse screening techniques are significant for discovering new potential targets of existing drugs or active molecules and for dissecting their molecular mechanisms or side effects [16]. Reverse screening finds the unknown protein targets of a given compound or additional targets of existing drugs among a large number of receptors by analysing their known ligands or crystal structures. It can be divided into three major groups: shape screening, pharmacophore screening and reverse docking [17]. When there is no receptor three-dimensional structure, shape or pharmacophore, screening allows the detection of the probable targets of a query molecule; this is done by comparing the overall shape or key pharmacophoric features of the query molecule with those of ligands included in a database annotated with information on their known targets [18,19,20]. The resulting targets can then be considered potential targets of the query molecule. Reverse docking refers to the successive docking of a query molecule into the active pocket of each potential target whose three-dimensional structure is known [21].

In this work a computational approach has been used to predict the potential molecular targets and the toxicological and/or side effects of BHT. The predicted interactions have been subsequently supported by molecular docking and analysed in light of the available literature data concerning the side effects of this antioxidant. 

## 2. Results

### 2.1. Target Prediction

The Swiss Target Prediction web tool was employed for the identification of potential targets of BHT, taken as “query” molecule. This tool estimates the chemical similarity (2D) and/or structural similarity (3D) of the query molecule with 376,342 compounds targeting 3068 different proteins [22]. The prediction was performed restricting the search to human proteins. Table 1 lists the predicted targets names ranked according to their score; targets having probability values under the threshold value of 0.3 are not listed.

Human Carbonic anhydrases II (CA2) is a cytosolic monomeric protein that catalyses the hydration of carbonic acid [23]. It plays a key role in human physiology; CA2 is involved in important physiological and pathologic processes; in particular, it is crucial in keeping the adequate balance between carbon dioxide and bicarbonate controlling pH and CO_2_ homeostasis, calcification, bone resorption, tumorigenicity, and many other processes [24]. In the literature, it is already known that CA2 binds BHT [24]. Thus, CA2 was excluded from further analyses.

The γ-Aminobutyric acid type A (GABA-A) receptor represents the major inhibitory neurotransmitter receptor [25] in the central nervous system (CNS). GABA-A receptors (GABA-ARs) make up a family composed of receptor subtypes in turn constituted by the combination of 19 different subunits. The subunits are classified into α, β, γ, δ, ε, and σ according to sequence similarity [26], while most GABA-AR are pentameric complexes (e.g., 2α, 2β, 1γ). Each subunit has a similar topology, with four membrane-spanning alpha-helical domains (M1–M4) [26].

Serotonin (5-hydroxytryptamine; 5-HT) is an endogenous monoamine neurotransmitter. Its signalling is mediated by seven different classes of receptors (5-HTRs), located both in CNS and periphery [27]. All 5-HTRs except 5-HT_3_R subtype belong to the class of the G-protein-coupled receptors (GPCRs) and are involved in many CNS processes, including the modulation of behaviour, mood, aggression, anxiety, and nociception [28,29,30,31].

The fatty acid ciclooxygenase 1 (COX-1) is a bifunctional, membrane-bound enzyme that catalyzes the dioxygenation of arachidonic acid to form prostaglandin H2 [32].

The human sodium-dependent noradrenaline transporter (NET; SLC6A2) belongs to the Na^+^/Cl^−^ dependent monoamine transporter family. It is involved in the recapture of norepinephrine (NE) neurotransmitter [33,34].

Noteworthy, the compound most similar to BHT (in 2D and 3D) known to be bioactive resulted to be propofol (2,6-diisopropylphenol) (PFL), which is an intravenous sedative-hypnotic agent employed for anaesthesia and sedation [35]. Interestingly, PFL is also known to be a potent antioxidant, as BHT, with anti-inflammatory and bronchodilator properties [36]. 

ADME analysis, performed using SwissADME, predicted that BHT is able to be assimilated by the gastrointestinal tract and to penetrate the blood–brain barrier (BBB) (Supplementary Figure S7; see Supplementary Materials for method details) [37]. Therefore, in principle, all the identified targets can be reached by BHT. 

### 2.2. Molecular Docking Studies

Docking simulations, carried out using AutoDock Vina through the DockingApp’s interface [38], were used to predict the binding mode of BHT (and of the similar compound PFL) to the above-mentioned targets. AutoDock Vina score values obtained by docking simulations, are reported in Table 2.

#### 2.2.1. GABA-A 

**Figure 1:**
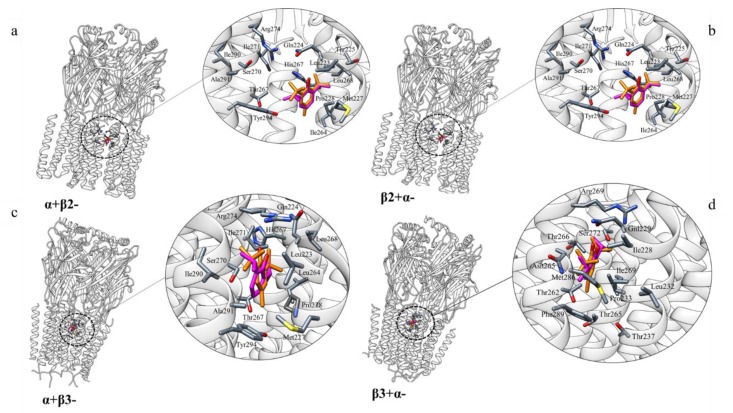
Molecular docking results for the GABA-AR–BHT interaction and the GABA-AR–PFL interaction (BHT and PFL are coloured in orange and magenta, respectively; protein residues are coloured by atom type); α1β2γ2 subtype, panels (**a**,**b**); α1β3γ2 subtype, panels (**c**,**d**).

A docking simulation study was carried out to analyse the interaction between BHT and GABA-AR, which is an important pharmacological target that can be activated and modulated by PFL [39,40]. Where and how PFL binds to GABA-AR is not clear as multiple binding sites have been detected [41]. In this context, studies have identified several amino acid residues in the α and β subunit transmembrane domains that seem to be important for the action of this anaesthetic drug. Based on these data, BHT molecule was docked onto the three-dimensional (3D) structures of α1β2γ2 (PDB 6D6U) and α1β3γ2 (PDB 6HUJ) GABA-AR subtypes. The molecular docking results, obtained using AutoDock Vina (see Methods for details), indicated a very similar binding mode for PFL and BHT. Common residues involved are (M1, M2 and M3 in parentheses indicating the number of the transmembrane helical domain): Ile228(M1), Gln229(M1), Leu232(M1), Pro233(M1), Thr237(M1), Thr262(M2), Asn265 (M2), Thr265(M2) Thr266(M2), Arg269(M2), Leu269(M2), Ser272(M2), Met286(M3), Phe289(M3) on the α-β+ interface (Figure 1b,d); Leu223(M1), Gln224(M1), Met227(M1), Pro228(M1), Ile264(M2), His267(M2), Thr267(M2) Leu268(M2), Ser270(M2), Ile271(M2), Arg274(M2), Ala291(M3), Tyr294(M3), on α+β- interface (Figure 1a,c).

#### 2.2.2. 5-HT_2B_ and 5-HT_2C_ Receptors

**Figure 2:**
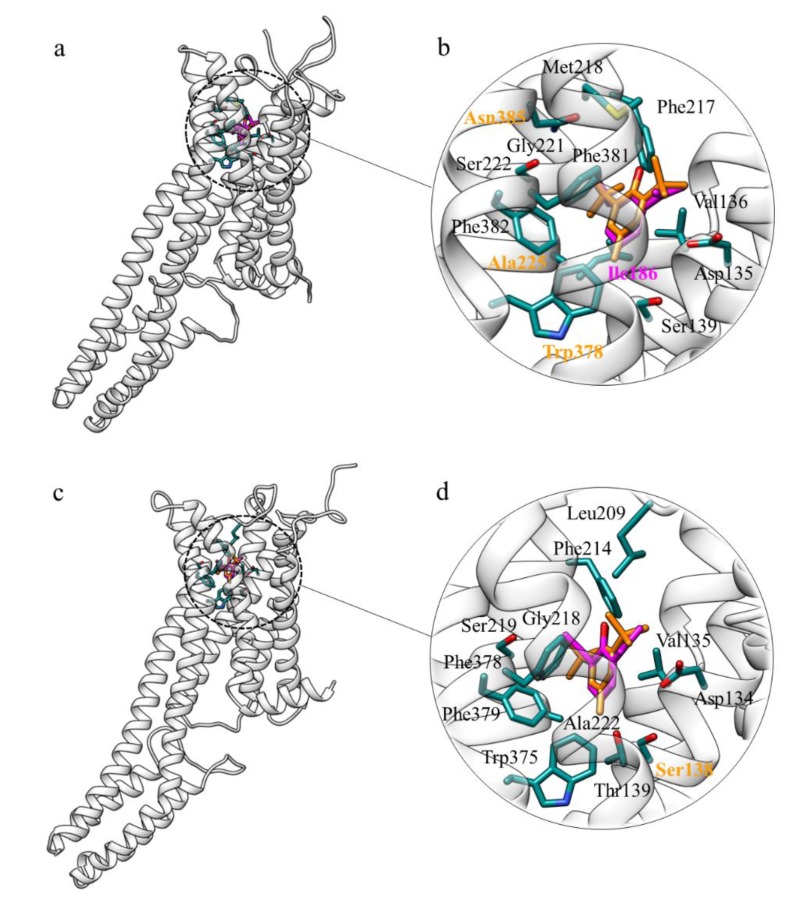
(**a**) Superimposition of the top-scoring poses for BHT (in orange) and PFL (in magenta) binding to 5-HT_2B_R obtained by docking simulations; (**b**) detail of the hydrophobic interactions between 5-HT_2B_R residues (coloured by atom type, carbon atoms in dark cyan) and the two ligands: BHT (in orange) and PFL (in magenta). The labels of the 5-HT_2B_R residues interacting with both BHT and PFL are coloured in black. Asn385, Ala225, Trp378 (depicted in orange) interact only with BHT, while Ile186 (in magenta) only with PFL; (**c**) Superimposition of the top-scoring poses for BHT (in orange) and PFL (in magenta) binding to 5-HT_2C_R; (**d**) detail of the hydrophobic interactions between 5-HT_2C_R residues (coloured by atom type, carbon atoms in dark cyan, black labels) and the two molecules, BHT (in orange) and PFL (in magenta). Ser138 interacts only with BHT, while the other residues with both ligands.

In order to further analyse if the serotonin receptors 5-HT_2B/2C_ can be targets of BHT, molecular docking simulations between the three-dimensional structures of the two receptors (PDB codes: 4IB4 and 6BQG) and BHT have been performed on the basis of the data obtained by Matsunaga and co-workers [42]. In the top-predicted binding poses (Figure 2a–c), BHT is located in the orthosteric site, or the 5-HT binding site, like PFL. Known 5-HT receptors ligands bind through interaction with Asp135 and with Ser222. Asp135 is a highly conserved residue among serotonergic receptors and it is believed to act as a counter-ion for the protonated amine of various ligands, while Ser222 provides a second interaction site for the protonated amine of serotonin [43]. However, BHT lacks this amine moiety and therefore binds to 5-HT_2B/2C_ in different ways. Indeed, the 5-HT_2B_R-BHT and 5-HT_2C_R-BHT complexes are stabilized by hydrophobic interactions with Asp135, Val136, Ser139, Phe217, Met218, Gly221, Ser222, Ala225, Trp378, Phe381, Phe382, Asn385 (Figure 2b) and with Asp134, Val135, Ser138, Leu209, Phe214, Gly218, Ser219, Ala222, Trp375, Phe378 and Phe379 (Figure 2d), respectively. Notably, residues Trp378, Phe382 in 5-HT2BR and Trp375, Phe379 in 5-HT_2C_R are believed to be involved in anchoring the aromatic moiety of the ligands [44] and, in the predicted complexes, the aromatic ring of BHT is close to these aromatic residues (Figure 2b,d). The superimposition of the top-predicted poses of BHT with those obtained for PFL (Figure 2) shows that the two molecules bind in the same region, establishing hydrophobic interactions with similar residues (Figure 2b,c). Further, the score value derived from docking simulations for PFL (Table 2) well correlates with the affinity value obtained experimentally by Matsunaga and co-workers [42]. BHT is predicted to bind with a score even higher, at least to the 5-HT_2C_ receptor, providing a strong support to the SwissTargetPrediction results.

#### 2.2.3. Cyclooxygenase 1 (COX-1)

**Figure 3:**
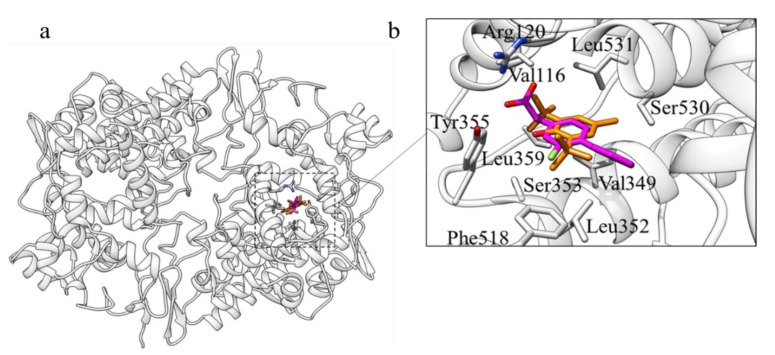
The best docking pose for BHT (orange) binding to COX-1 superimposed with the co-crystallized ligand, FLP (magenta). Protein residues are coloured by atom type, carbon atoms in grey. (**a**) Overall view; (**b**) detail of the binding pocket.

Another potential target of BHT identified in this study is the human cyclooxygenase 1 (COX-1) enzyme. There is currently no available crystal structure of this protein; for this reason, the structure of *Ovis aries* COX-1 (PDB ID: 1CQE) has been used as a template for structure modelling and further analysis of the human counterpart. The alignment of the two sequences with BLAST revealed a similarity of 92.59% with a coverage of 96%. Moreover, 1CQE was crystallized with flurbiprofen (FLP), a potent COX-1 inhibitor. Interestingly, in the blind docking simulation carried out on the structural model of human COX-1, BHT was located inside the FLP binding site (Figure 3). Additional simulations focussed on the FLP binding pocket resulted in a similar orientation of BHT with respect to FLP. Noteworthy, crucial interactions for the inhibitory action of FLP were maintained in the predicted complex with BHT (Figure 3b). Indeed, after the superimposition of the crystallized ligand with the docked one, the hydroxyl group of BHT was in spatial proximity of the carboxyl group of FLP. In addition, the ring of BHT is partially superimposed to that of FLP, being also in contact with the same residues, namely: Val349, Val116, Leu531, Leu352, Leu359, Ser353, Ser530, Tyr355, Phe518, Ala527. In particular the hydrogen bond with Tyr355, which plays a pivotal role in the inhibition of COX-1 by FLP together with Arg120 in the constriction site of the binding pocket [45], appears to be retained in the BHT predicted complex.

#### 2.2.4. Sodium-Dependent Noradrenaline Transporter (hNET, SLC6A2)

The human noradrenaline transporter (hNET; SLC6A2) is another predicted target of BHT. Additionally, in this case, to support the prediction, molecular docking simulations have been performed. Since no crystal structure is available for hNET, the corresponding protein sequence was submitted to a BLAST search against the Protein Data Bank (PDB) database, in order to retrieve the most similar proteins with a known structure. These resulted to be the human serotonin transporter (hSERT, PDB ID: 5I6X), and the *Drosophila melanogaster* dopamine transporter (dDAT, PDB ID: 4XP9). A structural model of hNET was thus built using the I-TASSER webserver. All the three structures were used as receptors for molecular docking simulations, with PFL and BHT as ligands, in order to compare the simulations results and to reinforce the reliability of the predictions. BHT and PFL established similar hydrophobic interactions in all the different docking simulation.

**Figure 4:**
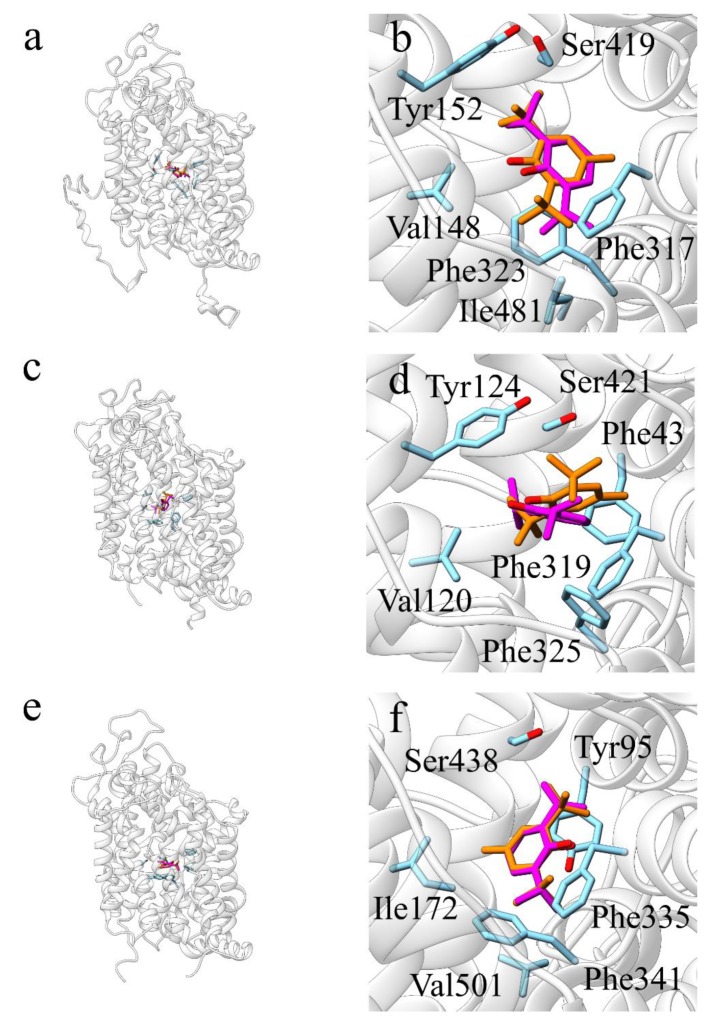
Depiction of the best poses for BHT and PFL binding to hNET, dDAT and hSERT obtained by docking simulations. The best docking poses for BHT (orange) and PFL (magenta) are illustrated in (**a**,**b**) for hNET, in (**c**,**d**) for dDAT, and in (**e**,**f**) for hSERT, respectively. Interacting residues are shown as stick (coloured by atom type, carbon atoms in cyan).

In detail, when the I-TASSER model of hNET was used as receptor, both PFL and BHT, with AutoDock Vina scores of −6.8 and −7.4 (Table 2), established hydrophobic interactions with Val148, Tyr152, Phe317, Gly320, Phe323, Ser419 and Ile481 (Figure 4a,b); when dDAT was used as receptor, both PFL (−5.9) and BHT (−6.5) established hydrophobic contacts with Phe43, Val120, Tyr124, Phe319, Phe325 and Ser421 (Figure 4c,d); when hSERT was used as receptor, both PFL (−6.8) and BHT (−7.6) established hydrophobic interactions with Tyr95, Ile172, Phe335, Gly338, Phe341, Ser438, Val501 (Figure 4e,f).

In all three cases, binding of the ligands is predicted to be stabilized by π-stacking interactions of their aromatic ring with phenylalanine residues: Phe323 in hNET, Phe325 in dDAT and Phe341 in hSERT.

### 2.3. Virtual Screening against ChEMBL Compounds

In order to evaluate the significance of these results, a comparison of the AutoDock Vina score of BHT with a reference scale was carried out. The reference scale is composed by the scoring values predicted by means of AutoDock Vina on a selection of compounds experimentally tested against the identified targets, retrieved from ChEMBL [46]. A detailed description of the methodology and the results of this analysis is reported in the Supplementary Materials.

Surprisingly, the results of this analysis indicate that the correlation between AutoDock Vina score values and experimental binding affinity values is at most very weak. With these caution in mind, the analysis reveals that in most of the cases the AutoDock Vina scores for BHT is higher than that calculated for many weak binders and some strong binders as well (Supplementary Figures S2–S4 and S6). This is especially true for GABA-AR α-1/β-3/γ-2 α+β3- interface (Supplementary Figure S2) and for 5-HT_2C_R (Supplementary Figure S4). Conversely, in the case of the α+β2- interface of GABA-AR α-1/β-2/γ-2 and COX-1, the predicted score value for BHT falls below the score distribution of the reference scale for both strong and weak binders (Supplementary Figures S1 and S5, respectively). According to this analysis and taking into account the weak correlation observed, the two latter targets must definitely be considered low confidence ones.

## 3. Discussion

In the present work, it has been probed the usefulness of reverse screening and molecular docking techniques for the identification of secondary targets of a commonly used food additive, i.e., BHT. This synthetic phenolic antioxidant is widely used as an additive in the food, cosmetic, and plastic industries [9]. In order to investigate the potential effects of human long-term exposure, a great number of toxicity studies have been carried out [47,48,49,50,51]. In this context, controversial results regarding the toxicity of BHT and its metabolites have been reported. Indeed, most of the studies were accomplished using animal models, making it difficult to translate the results to humans, due to different exposure conditions and metabolites generation. Starting from SwissTargetPrediction results, four different targets were selected: GABA-A receptor, 5-HT_2B-2C_R, COX-1 and hNET. To validate these findings, molecular docking simulations were also carried out. 

### 3.1. GABA-A Receptor

GABA-ARs are members of the Cys-loop superfamily of ligand-gated ion channels. Each subtype, determined by the subunits composition, has distinct anatomical and subcellular localizations. It mediates the fast-inhibitory neurotransmission in CNS; dysfunction of this receptor results in neurological and mental disorders such as epilepsy, anxiety and sleep impairment, problems in learning, memory and sensorimotor processing, and neurodevelopmental disorders, including autism [52,53,54,55]. Alongside GABA, this superfamily comprises both cationic nicotine and 5-HT (5-hydroxytryptamine) receptors, and glycine receptors ion channels [56]. GABA-A receptors are the targets of a diverse set of drugs which act via different binding sites. The great pharmacological variety of this receptor is related to its complex subunit organization. To identify the BHT-binding region, residues known to be important for PFL binding to GABA-AR, have been selected [41,57,58]. Binding sites for BHT have been found at both β+α- and α+β- interfaces. Studies have suggested markedly weaker PFL binding to the α+γ and γ+β-; thus, those interfaces were not investigated in this work [59]. 

The docking results of α1β2γ2 isoform differ slightly from α1β3γ2 type, in particular the unusual values of AutoDock Vina score obtained from the β3+α1- simulation (Table 2, Figure 1d) may be due to the fact that in the structure of this receptor subtype the orientation of the α and β subunits is different from that observed in other structures; in particular, the α1β3γ2 subtype adopts a conformation in which the receptor channel is closed [60]. It is worth noting that α1 Ser270(M2) and α1 Ala291(M3) residues are part of the binding site for ethanol [61], halothane, isoflurane, and PFL [62,63]. Mutation of these residues greatly decreases positive modulation of GABA-AR by small molecules [64,65,66]. It has been demonstrated that residues β2/3-Asn265(M2) and Met286 (TM3), homologous to α1 S270(M2) and α1 A291(M3), are essential for receptor modulation by general anaesthetics and anticonvulsants [26,57]. In addition, the mutants His267Ala and Met227Trp caused changes in GABA sensitivity to some of the anaesthetics [59]. These data support the crucial role of Asn265, Met286, His267, Ala291, Ser270 and Met227 in GABA-AR drug-mediated modulation [67]. Thus, docking simulations, predicting BHT interaction with these residues, suggest that it could allosterically modulate GABA-ARs and disrupt normal physiologic circuits.

### 3.2. 5-HT_2B-2C_R

5-HT_2_Rs subtypes of the serotonin receptors family belong to the class of the G-protein-coupled receptors coupled to Gαq/11, whose signalling leads to numerous physiological responses, such as fertilization, cell growth, transformation, secretion, smooth muscle contraction, sensory perception and neuronal signalling [43,68,69,70,71,72,73,74,75,76]. Signalling of 5-HT is mediated by seven different classes of receptors, located both in the CNS and in the periphery. The 5-HT_2_ receptor (5-HT_2_R) family consists of three isoforms (5-HT_2A_R, 5-HT_2B_R, 5-HT_2C_R) which share ~50% total sequence similarity and ~80% sequence similarity within their seven transmembrane domains [77]. 

Several studies have investigated the functional role of the 5-HT_2B_R in the regulation of peripheral functions and have found that this receptor is involved in the control of cell differentiation and proliferation, and in the regulation of gastrointestinal, vascular, pulmonary and cardiac functions [78,79,80,81,82,83,84,85,86]. However, the functional role of 5-HT_2B_Rs within the central nervous system (CNS) has been poorly analysed [87]. In turn, 5-HT_2C_R is believed to have an important function in the control of many physiological and behavioural responses, including feeding, anxiety, temperature regulation, locomotion, sexual behaviour and the occurrence of seizures, in addition to a role in mood disorders [88,89].

Most of the studies concerning the interactions between anaesthetic agents and the 5-HT receptors have mainly focused on the 5-HT_3_ class, which includes ligand-gated excitatory ion channels and is involved in the modulation of nausea and vomiting [42]. 5-HT_3_ receptors are depressed by the anaesthetic PFL, which, in contrast, enhances the activity of inhibitory ligand-gated ion channels such as γ-aminobutyric acid type A receptors and glycine receptors. 

Except for some studies that have shown evidence for the interaction between anaesthetics and 5-HT_2A_ and 5-HT_3_ at millimolar concentrations, no other studies have investigated if any other 5-HT receptors interact with general anaesthetics at pharmacologically relevant concentrations [42]. Only Matsunaga and co-workers have carried out both computational and experimental studies to investigate the molecular interactions between PFL and 5-HT_2B_Rs. In particular, they have performed radioligand binding screening on the serotonin receptors, providing insights into how PFL and other anaesthetics interact with 5-HT_2B_ receptor.

Therefore, in this work molecular docking simulations between 5-HT_2B-2C_R and BHT have been performed based on the data obtained by Matsunaga. The results show that BHT and PFL are located in the same binding site and establishing hydrophobic interactions with the same 5-HT_2B-2C_R residues (Figure 2). In particular, PFL and BHT binding occurs by interactions between the phenyl ring and the aromatic residues Trp378, Phe381, and Phe382 of 5-HT2BR and Trp375, Phe378, and Phe379 of 5-HT_2C_R. Interestingly, this aromatic pattern is conserved between the three 5HT_2_ receptor subtypes. Since GPCRs are anaesthetics targets [90,91] and that PFL and BHT are similar compounds, these two might interact directly with 5-HT_2B_-_2C_R, thus modifying their function through either agonist or antagonist modes of action. 

### 3.3. COX-1

There are two distinct isoforms of COX. COX-1 is found on the inner and outer membrane of the nuclear envelope and in the luminal surfaces of the endoplasmic reticulum. It is constitutively expressed, producing prostaglandins that mediate the housekeeping functions of the organism. Conversely, COX-2 is induced in response to cytokines, endotoxins and tumour and is present in only a few tissues. COXs are the principal target of nonsteroidal anti-inflammatory drugs (NSAID): COX-2 is involved in the analgesic and anti-inflammatory effect of NSAIDs, while inhibition of COX-1 is responsible for the ulcerogenic side effect of such drugs [92].

Various works reported that prolonged BHT administration induces a chronic inflammatory response characterized by increased macrophage infiltration, vascular leakage, and elevated COX-1 and COX-2 expression [93]. At the same time, PFL was observed to reduce both the activity and the expression of COX [94]. However, there are no proofs of a direct interaction of BHT and COX. In order to elucidate the mechanism of interaction between cyclooxygenase and BHT, molecular docking simulations were carried out. The BHT interaction profile was similar to the one of FLP, a known non-reversible inhibitor of COX-1 [45]. Indeed, the aromatic ring of both BHT and FLP are partly superimposed and in contact with Val359 and Leu352. Furthermore, a crucial interaction, responsible of the inhibitory effect of FLP, is partly conserved. This interaction involves the oxydryl group of BHT (carbonyl-group in the case of FLP) with Arg120 and Tyr355 of the constriction site of COX-1. However, the oxydryl group of BHT is located too far away from Arg120 sidechain due to geometric constrains. To better explore the possibility of an interaction between BHT and Tyr355 and Arg120, a flexible docking has been performed. This allows the Arg120 side chain to get closer to the oxydryl group of BHT, as shown in Figure 3.

### 3.4. NET

Mutations of the NET gene have been correlated to orthostatic intolerance [95] and it has been hypothesized that they could have a role in the anorexia nervosa disorder [96]. Moreover, this protein is also a target of several antidepressant drugs, such the reboxetine [97].

The molecular docking analyses pointed out a similar interaction pattern for BHT and PFL, in hNET, dDAT, and hSERT. Of note, the anaesthetic PFL has been shown to have an inhibitory activity on hNET at a clinically relevant concentration [98]. Moreover, the ligand–residues interactions predicted through molecular docking simulations are consistent with the binding mode of inhibitors and substrates observed in the crystallized structures of dDAT and hSERT [98,99,100]. In particular, the contacts established with Val, Tyr, Phe and Ser residues are important for the binding of the co-crystallized ligands. These results suggest that BHT could have an inhibitory activity toward NET and the other monoamine transporters (SERT and DAT), as shown for PFL [98,101]. However, it should be noted that in two previous works, BHT and PFL were responsible for the modulation of DAT and NET expression, implying that the activity of these two molecules could be the result of both a direct and an indirect effect on these neurotransmitter transporters [10,102].

## 4. Methods 

### 4.1. SwissTarget Prediction 

Putative secondary targets for BHT have been identified by using SwissTargetPrediction (available at http://www.swisstargetprediction.ch/): γ-Aminobutyric acid type A (GABA-A) receptor, Serotonin 2b and 2c receptors, the fatty acid ciclooxygenase 1 (COX-1), the human sodium-dependent noradrenaline transporter (NET; SLC6A2). It calculates the similarity between the input compounds and those contained in a curated collection correlated with the data provided by experimental binding assays. The similarity between two small molecules is calculated based both on 2D and 3D metrics. For the 2D measurement, the molecules are encoded through a binary fingerprint (FP2) [103] and the evaluation metrics employed is the Tanimoto’s coefficient. The 3D similarity is calculated by using the Manhattan distance between the molecules’ shapes encoded using Electroshape 5D (5ES5D) [104,105]. This method relies on a five-dimensional representation of the molecule, adding to the Cartesian coordinates the partial charges and lipophilic contribution. Based on the three-dimensional structure of the molecule, four representative points are identified. From each of these points, called centroids, the distribution of the distances from every atom of the molecule are calculated [22]. After the similarity search is completed, a confidence score is assigned to each prediction.

### 4.2. Molecular Modeling 

The structure prediction of the hNET protein has been carried out through I-TASSER (https://zhanglab.ccmb.med.umich.edu/I-TASSER/) server pipeline [106]. 

The two missing regions within 5-HT_2B_-_2C_R structure (PDB codes: 4IB4 and 6BQG) have been modelled with MODELLER [107,108]. 

### 4.3. Molecular Docking

Molecular docking simulations have been performed using AutoDock Vina [109] through the DockingApp’s interface [38]. Docking simulations have been performed between BHT/PFL and GABA-A receptors (PDB codes: 6HUJ, 6D6U), 5-HT_2B-2c_R (PDB codes: 4IB4 and 6BQG), COX1 (PDB code: 1CQE) and NET (hNET: model, hSERT, PDB code: 5I6X; dDAT, PDB code: 4XP9). Docking simulations have also been performed between BHT/FPL and COX-1 (PDB code: 1CQE). BHT’s conformation has been generated starting from the molecule’s SMILES using ETKDG [110], a conformer generation method implemented in RDKIT (Open-source cheminformatics; http://www.rdkit.org). This method combines a distance geometry approach with knowledge of preferred torsional angles, derived from experimentally determined crystal structures, and with constraints from chemical knowledge (e.g., bonds connected to triple bonds are colinear, aromatic ring must be flat). The protein and the co-crystallized ligands have been prepared with AutoDock Tools [111] by merging nonpolar hydrogens and adding Gasteiger partial charges. The grid box has been centred for each target on the native binding site.

## 5. Conclusions

In conclusion, the results here described reveal common interaction patterns within all the structures analysed: several hydrophobic interactions stabilize BHT in all targets, whereas in GABA-AR and COX-1, hydrogen bonds interactions are also conserved (i.e., Asn265 and Tyr355, respectively). The identified interactions are also established by PFL and other co-crystallized inhibitors (see COX-1). Noteworthy, the pivotal role played by the detected residues is supported by experimental data available in the literature [44,45,67,100]. According to the results of this work, side effects of BHT, such as anxiety [10] and reduced heart beating activity [48], could be caused by modulation of 5-HT_2C_, GABA-AR, NET and DAT, in the central nervous system. This hypothesis is also supported by ADME analysis indicating that BHT is able to cross the blood–brain barrier.

It is worthwhile to remark that these conclusions must be taken with caution as the virtual screening analysis did not evidence a clear correlation between AutoDock Vina score values and experimentally determined binding affinity values. This is probably due in part to the approximate nature of the AutoDock Vina scoring function and in part to the different techniques and experimental conditions (pH, buffer components, etc.) used to obtain the binding affinity data retrieved from ChEMBL database. Thus, further experimental analyses are certainly required to support these findings.

Finally, the results reported in this work confirm the usefulness of computational approaches in the identification of potential secondary targets responsible for undesired side effects of food additives, identification that can aid in the subsequent experimental work.

## Figures and Tables

**Table 1:** Results of Swiss Target Prediction.

Predicted Target	Probability Value
Carbonic anhydrase II	1.00
GABA-A receptor; α-1/β-2/γ-2	0.61
Serotonin 2b receptor	0.37
GABA-A receptor; α-1/β-3/γ-2	0.37
Cyclooxygenase-1	0.37
Norepinephrine transporter	0.37
Serotonin 2c receptor	0.37

**Table 2:** AutoDock Vina scores obtained by docking simulations.

Target.	AutoDock Vina Score		
	BHT	PFL	FLP ^a^
GABA-AR α+β2- ^b^	−5.6	−5.3	
GABA-AR β2+α-	−6.6	−6.1	
GABA-AR α+β3-	−5.3	−6.6	
GABA-AR β3+α-	1.5	−2.5	
5-HT_2B_R	−6.3	−6.5	
5-HT_2C_R	−7.0	−6.3	
COX-1	−6.1		−8.5
hNET	−7.4	−6.8	
dDAT	−6.5	−5.9	
hSERT	−7.6	−6.8	

^a^ For GABA-AR the notation α+β2-, β2+α-, α+β3-, β3+α- refers to the different intersubunits interfaces. ^b^ For COX-1 docking simulations were performed using BHT and flurbiprofen (FLP), instead of PFL, as ligands. The results obtained for each target is hereby described.

## Supplementary Materials

The following are available online, Table S1: Total number of compounds used for the virtual screening against each target, Figure S1: Virtual screening results for α+β2- (above) and α-β2+ (below) interfaces of the GABA-AR α-1/β-2/γ-2, Figure S2: Virtual screening results for α+β3- interface of the GABA-AR α-1/β-3/γ-2, Figure S3: Virtual screening results for 5-HT_2B_R, Figure S4: Virtual screening results for 5-HT_2C_R, Figure S5: Virtual screening results for COX-1, Figure S6: Virtual screening results for hNET, Figure S7: ADME analysis performed with SwissADME.
