# Supplementary material for: Computational Methods for the Identification of Molecular Targets of Toxic Food Additives. Butylated Hydroxytoluene as a Case Study
Source: Molecules. 2020 May 9;25(9):2229. doi: 10.3390/molecules25092229 (PMC7248939; doi:10.3390/molecules25092229)
Supplement: Supplementary file 1 [file molecules-25-02229-s001.pdf]

Computational methods for the identification of molecular targets of toxic food additives. Butylated hydroxytoluene as a case study

Valentina Tortosa[1], Valentina Pietropaolo[1], Valentina Brandi[1], Gabriele Macari[1], Andrea Pasquadibisceglie[1], and Fabio Polticelli [1, 2,*]

[1]Department of Sciences, Roma Tre University, 00146 Rome, Italy

[2]National Institute of Nuclear Physics, Roma Tre University, 00146 Rome, Italy

*Correspondence: Prof. Fabio Polticelli; fabio.polticelli@uniroma3.it; Tel.: +39-06-5733-6362 (F.L.)

Academic editor:

Received: date; Accepted: date; Published: date

Supplementary Methods

1. ChEMBL compounds virtual screening

The list of tested compounds has been retrieved from ChEMBL using the official python client named *ChEMBL webresource client* [1]. The ChEMBL IDs used were: CHEMBL221 for hNET; CHEMBL222 for COX-1; CHEMBL2095172 for GABA-AR α-1/β-2/γ-2 receptor; CHEMBL2094121 for GABA-AR α-1/β-3/γ-2 receptor; CHEMBL225 for 5-$HT_{2C}R$; CHEMBL1833 for 5-$HT_{2B}R$. Where possible *Ki* data were used, with the exception of COX-1 and GABA-AR α-1/β-2/γ-2 for which the IC50 values were used. Affinity values not expressed in nM were filtered out. For each target a sampling procedure of the compounds has been performed as follows: all affinity data were converted in *pKi*/*pIC50*; the data have been then divided into bins on the basis of the integer part of their value. For each bin, if possible, 30 compounds were randomly sampled. If a bin contained less than 30 compounds, all the compounds were taken instead. The total number of compounds for each target is reported in table S1. For each target-compounds pair library the docking simulations were performed using the same settings chosen for BHT and AutoDock Vina. Compounds were divided in strong binders if their *pKi*/*pIC50* values were greater than 6, otherwise they were defined weak binders. Due to the issues already discussed in the main text, GABA-AR β3+α- interface was not taken into consideration in these further tests. The results of this analysis are illustrated in Figures 1SM-6SM.

**Target**	**Compounds number**
GABA-AR α-1/β-2/γ-2	149
GABA-AR α-1/β-3/γ-2	75
5-$HT_{2B}R$	148
5-$HT_{2C}R$	165
COX-1	180
hNET	212

Table 1SM. Total number of compounds used for the virtual screening against each target.

2. ADME analysis

Assessment of absorption, distribution, metabolism and excretion (ADME) is a crucial part of drug development. Computational approaches can be used in place of experimental methodology to cut both time and cost. SwissADME is a web server for the prediction of small molecules physicochemical properties such as, but not limited to, pharmacokinetics properties and drug-likeness [2]. It employs several predictive models and when possible, it adopts a consensus approach (e.g. for lipophilicity prediction). In this work, ADME analysis has been performed in order to predict if BHT could reach the identified targets (Figure 7SM).

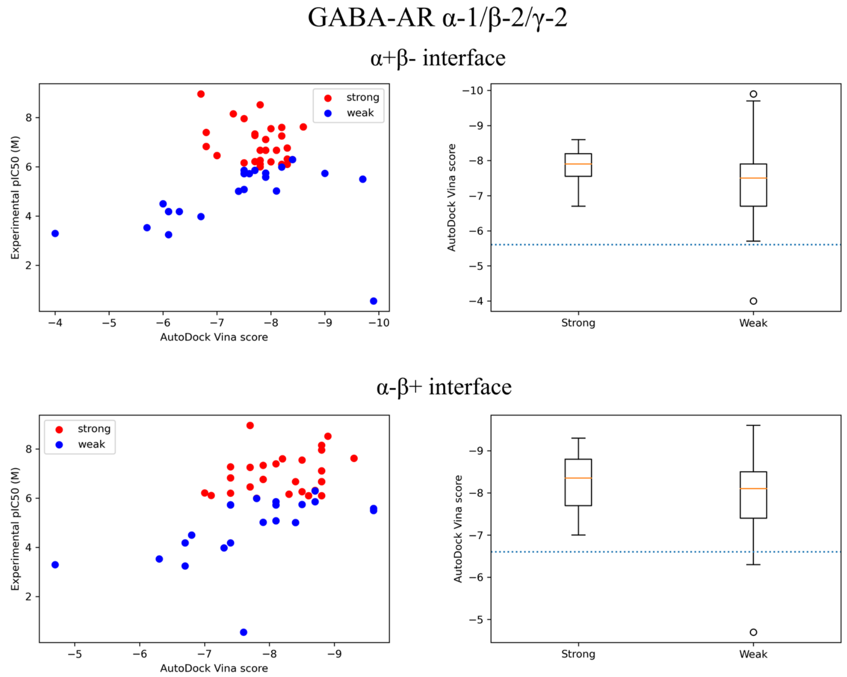

Figure 1SM. Virtual screening results for α+β2- (above) and α-β2+ (below) interfaces of the GABA-AR α-1/β-2/γ-2. In the scatterplots (on the left) are plotted the experimental results (*pIC50*) against the AutoDock Vina score results for the weak (blue) and strong (red) binders; in the box plots (on the right) are depicted the AutoDock Vina results distribution for strong and weak binders. The score for BHT (-5.6 kcal/mol for α+β2- and -6.6 kcal/mol for α-β2+) is represented as a dotted blue line.

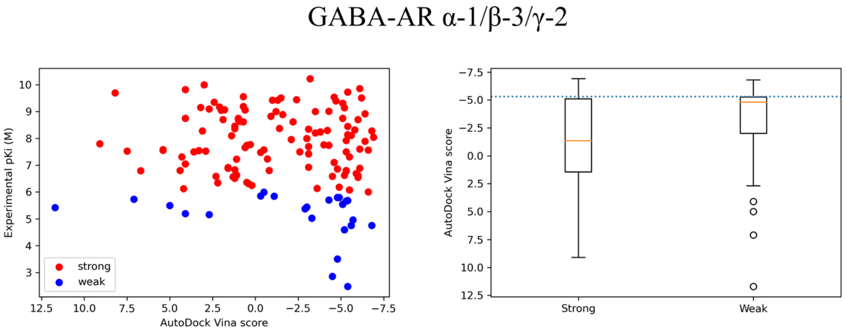

Figure 2SM. Virtual screening results for α+β3- interface of the GABA-AR α-1/β-3/γ-2. In the scatterplot (on the left) are plotted the experimental results (*pKi*) against the AutoDock Vina score results for the weak (blue) and strong (red) binders; in the box plot (on the right) are depicted the AutoDock Vina results distribution for strong and weak binders. The score for BHT (-5.3 kcal/mol for α+β3-) is represented as a dotted blue line.

5-HT2BR

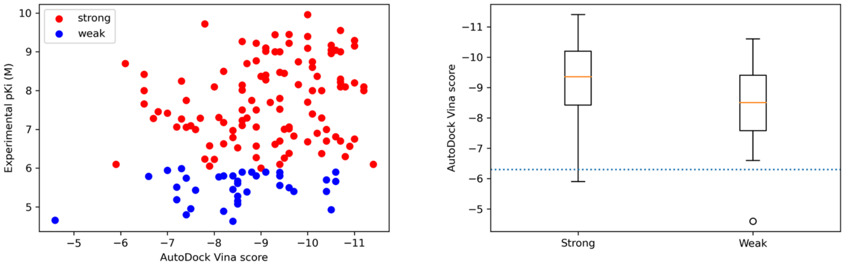

Figure 3SM. Virtual screening results for 5-HT$_{2B}$R. In the scatterplot (on the left) are plotted the experimental results (*pKi*) against the AutoDock Vina score results for the weak (blue) and strong (red) binders; in the box plot (on the right) are depicted the AutoDock Vina results distribution for strong and weak binders. The score for BHT (-7.0 kcal/mol) is represented as a dotted blue line.

5-HT2CR

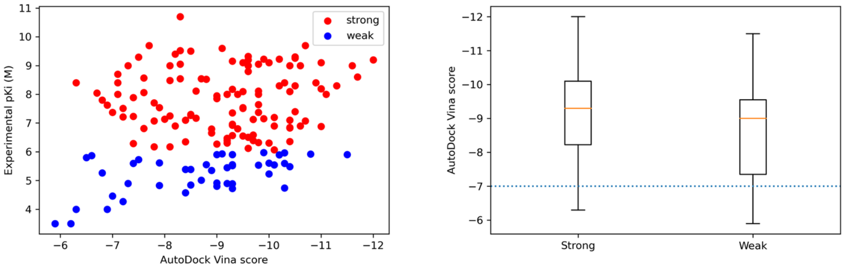

Figure 4SM. Virtual screening results for 5-HT$_{2C}$R. In the scatterplot (on the left) are plotted the experimental results (*pKi*) against the AutoDock Vina score results for the weak (blue) and strong (red) binders; in the box plot (on the right) are depicted the AutoDock Vina results distribution for strong and weak binders. The score for BHT (-7.7 kcal/mol) is represented as a dotted blue line.

COX-1

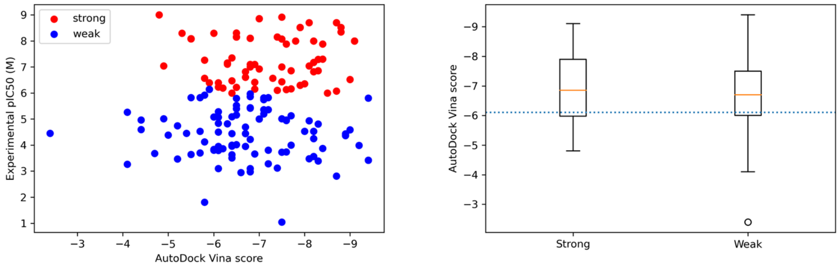

Figure 5SM. Virtual screening results for COX-1. In the scatterplot (on the left) are plotted the experimental results (*pIC50*) against the AutoDock Vina score results for the weak (blue) and strong (red) binders; in the box plot (on the right) are depicted the AutoDock Vina results

distribution for strong and weak binders. The score for BHT (-6.1 kcal/mol) is represented as a dotted blue line.

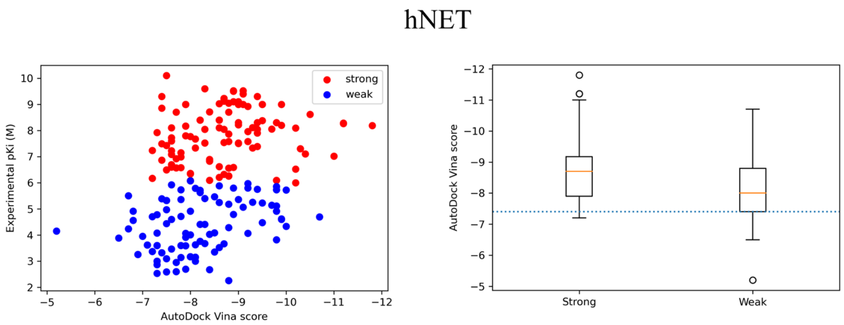

Figure 6SM. Virtual screening results for hNET. In the scatterplot (on the left) are plotted the experimental results (*pKi*) against the AutoDock Vina score results for the weak (blue) and strong (red) binders; in the box plot (on the right) are depicted the AutoDock Vina results distribution for strong and weak binders. The score for BHT (-7.4 kcal/mol) is represented as a dotted blue line.

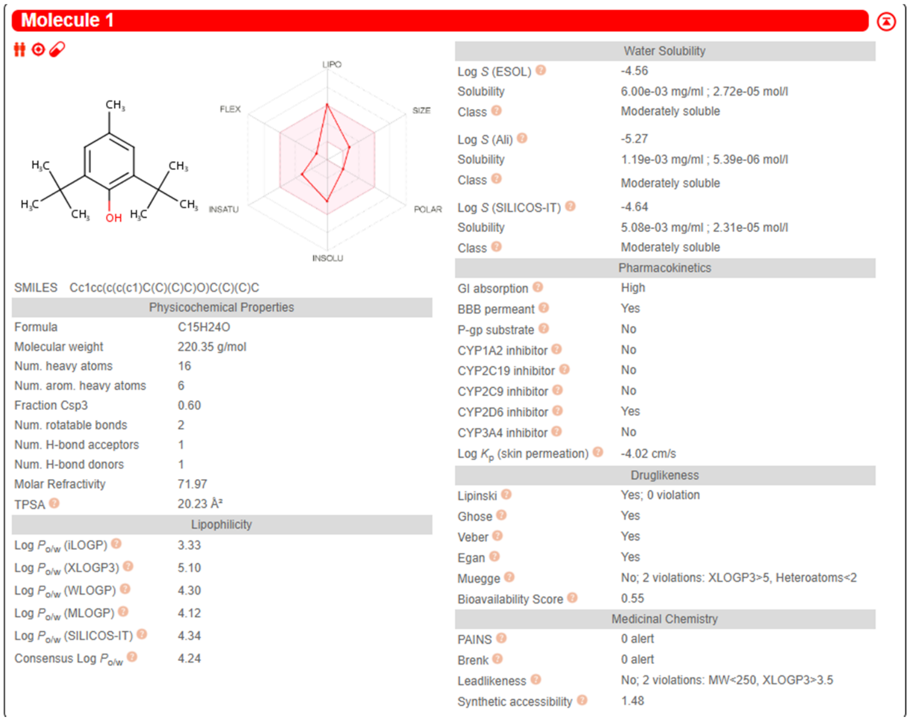

Physicochemical Properties	
Formula	C15H24O
Molecular weight	220.35 g/mol
Num. heavy atoms	16
Num. arom. heavy atoms	6
Fraction Csp3	0.60
Num. rotatable bonds	2
Num. H-bond acceptors	1
Num. H-bond donors	1
Molar Refractivity	71.97
TPSA	20.23 Å²
**Lipophilicity**	
Log $P_{o/w}$ (iLOGP)	3.33
Log $P_{o/w}$ (XLOGP3)	5.10
Log $P_{o/w}$ (WLOGP)	4.30
Log $P_{o/w}$ (MLOGP)	4.12
Log $P_{o/w}$ (SILICOS-IT)	4.34
Consensus Log $P_{o/w}$	4.24

Water Solubility	
Log *S* (ESOL)	-4.56
Solubility	6.00e-03 mg/ml ; 2.72e-05 mol/l
Class	Moderately soluble
Log *S* (Ali)	-5.27
Solubility	1.19e-03 mg/ml ; 5.39e-06 mol/l
Class	Moderately soluble
Log *S* (SILICOS-IT)	-4.64
Solubility	5.08e-03 mg/ml ; 2.31e-05 mol/l
Class	Moderately soluble
**Pharmacokinetics**	
GI absorption	High
BBB permeant	Yes
P-gp substrate	No
CYP1A2 inhibitor	No
CYP2C19 inhibitor	No
CYP2C9 inhibitor	No
CYP2D6 inhibitor	Yes
CYP3A4 inhibitor	No
Log K_p (skin permeation)	-4.02 cm/s
**Druglikeness**	
Lipinski	Yes; 0 violation
Ghose	Yes
Veber	Yes
Egan	Yes
Muegge	No; 2 violations: XLOGP3>5, Heteroatoms<2
Bioavailability Score	0.55
**Medicinal Chemistry**	
PAINS	0 alert
Brenk	0 alert
Leadlikeness	No; 2 violations: MW<250, XLOGP3>3.5
Synthetic accessibility	1.48

Figure 7SM. ADME analysis performed with SwissADME.

References

1. Gaulton, A., Bellis, L. J., Bento, A. P., Chambers, J., Davies, M., Hersey, A., Light, Y., McGlinchey, S., Michalovich, D., Al-Lazikani, B., & Overington, J. P. ChEMBL: a large-scale bioactivity database for drug discovery. *Nucleic acids res.* **2012**, *40(Database issue)*, D1100–D1107.

2. Daina, A., Michielin, O. & Zoete, V. SwissADME: a free web tool to evaluate pharmacokinetics, drug-likeness and medicinal chemistry friendliness of small molecules. *Sci Rep.* **2017**, *7*, 42717.
